# A Sweat Cortisol Sensor Based on Gold-Modified Molecularly Imprinted Polymer

**DOI:** 10.3390/nano15211654

**Published:** 2025-10-30

**Authors:** Ziyu Liu, Guangzhong Xie, Jing Li, Hong Yuan, Yuanjie Su

**Affiliations:** 1 State Key Laboratory of Electronic Thin Films and Integrated Devices, School of Optoelectronic Science and Engineering, University of Electronic Science and Technology of China, Chengdu 610054, China; 2 School of Automation Science and Electrical Engineering, Beihang University, Beijing 100191, China; 3 School of Architecture, Southwest Jiaotong University, Chengdu 611756, China

**Keywords:** cortisol sensor, sweat, electrochemistry, molecular imprinting, screen printing electrode

## Abstract

Approximately 3.8% of the global population suffers from depressive disorders, posing a substantial public health challenge exacerbated by the COVID-19 pandemic due to widespread unemployment and prolonged social isolation. The difficulty in objectively quantifying psychological states underscores the need for effective stress assessment methods. Herein, we developed a portable electrochemical cortisol sensor (PECS) for accurate mental stress assessment. The PECS consists of a screen-printed carbon electrode decorated with gold nanoparticles and a molecularly imprinted polymer (MIP) synthesized via electropolymerization. The as-prepared PECS demonstrates a wide and linear detection range from 1 fM to 1 μM, an ultra-low detection limit of 0.4112 fM and a high sensitivity of 15.518 nA∙lg(nM^−1^)∙cm^−2^. This work provides new possibility of developing soft bioelectronics for non-invasive and real-time mental health monitoring.

## 1. Introduction

Amidst the escalating global mental health crisis, depression, anxiety, and psychological stress pose significant challenges to public health—a concern that is particularly acute among students and young professionals. According to the “2022 National Depression Blue Book of China,” the lifetime prevalence of depression among Chinese adults reaches up to 6.8%, i.e., more than 95 million individuals. Furthermore, approximately 280,000 deaths by suicide are recorded annually, with 40% of these cases being closely associated with depression [1]. Globally, the number of mental-disorder patients exceed one billion [2]. The incidence of depression and associated disorders has been further exacerbated by the COVID-19 pandemic, posing unprecedented challenges to global mental health services [3,4].

**Figure 1:**
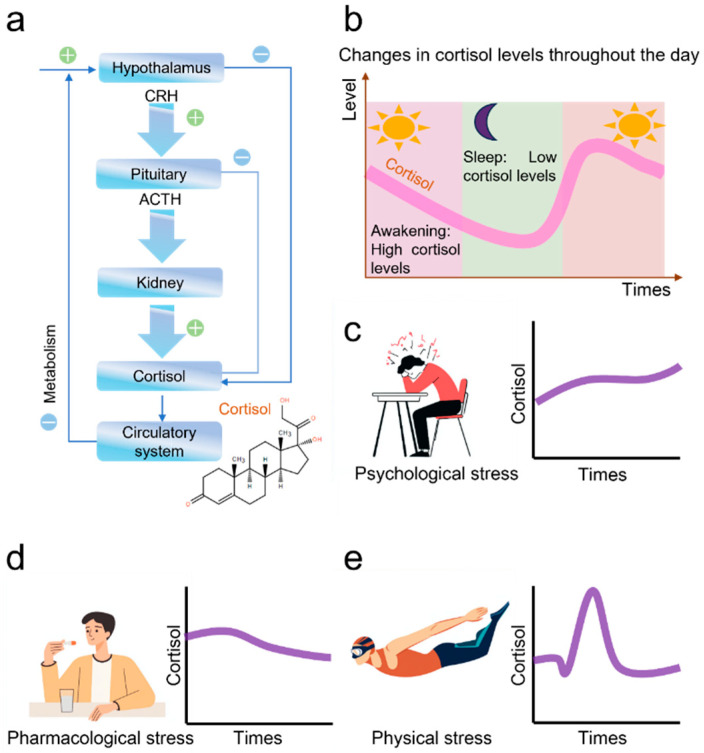
Cortisol Regulation and Stress Response. (**a**) The mechanism of cortisol production in the human body; (**b**) The diurnal rhythm of cortisol; (**c**) Changes in cortisol caused by psychological stress; (**d**) The effect of medication on cortisol levels; (**e**) The effect of exercise on cortisol levels.

Conventional methods like surveys and psychological tests lack the capacity of real-time and continuity, thereby compromising the accuracy and timeliness of assessments [5,6,7,8,9]. This highlights a compelling need for an efficient system capable of objective, real-time psychological stress monitoring. Cortisol, the principal hormone, demonstrates a strong correlation with psychological stress levels and serves as a key biomarker for evaluating psychological stress and depressive disorder [10]. Human biological response to stress is regulated by a complex feedback system known as the Hypothalamic–Pituitary–Adrenal (HPA) axis (Figure 1a). When the body is confronted with stress, the hypothalamus synthesizes and releases corticotropin-releasing hormone (CRH). This hormone then acts on the anterior pituitary gland, triggering it to secrete adrenocorticotropic hormone (ACTH) into the bloodstream. Subsequently, ACTH travels through the bloodstream to the adrenal glands and stimulates the secretion of hormones such as cortisol. The cortisol migrates through the lipid bilayer membrane into the circulatory system. Therefore, the concentration of cortisol in the blood is directly correlated with the amount of cortisol released by the adrenal glands.

Additionally, cortisol secretion follows a diurnal rhythm (Figure 1b) [11,12,13]: it peaks around 8 a.m. (50–230 ng/mL), and gradually decreases throughout the day, then reaches its lowest level in the evening (30–160 ng/mL). Under normal conditions, the HPA axis releases cortisol and related hormones during the acute phase of stress, in which the feedback system returns to baseline once the stressor is removed.

However, the chronic stress or prolonged mental strain would dysregulate the HPA axis, rendering abnormally elevated cortisol level (Figure 1c). Meanwhile, this can lead to increased inflammation, impaired immune function, metabolic disorders, depression, anxiety, cardiovascular disease, and other stress-related conditions. Conversely, medication-triggered psychological disorders lower the body’s cortisol levels over a period (Figure 1d), while strenuous exercises cause a transient increase in cortisol concentration (Figure 1e).

Conventional cortisol quantification methods (such as assays of blood, saliva, and urine) suffer from invasiveness, vulnerability, and discontinuity, and fail to meet the requirement for efficient and convenient monitoring in real time. In contrast, sweat-based cortisol sensing technology offers a promising non-invasive diagnosis platform with various distinct advantages like real-time data acquisition and high sensitivity. These merits enables real-time and continuous tracking of cortisol concentration and facilitates stress assessments with high-specificity and high-fidelity, suitable for personalized medicine and remote health monitoring [12,14,15,16,17].

A critical challenge in the development of such wearable sensors lies in the capability of accurately distinguishing the target analyte from the complex mixture of interfering substances in sweat. This high selectivity originates from a molecular recognition element integrated into the sensor platform, in which biological receptors, such as antibodies and aptamers, are commonly employed for this purpose. Nevertheless, the application of those receptors in wearable devices is often hindered by inherent limitations, including high production costs, limited shelf life, and susceptibility to denaturation under the variable environmental conditions (e.g., temperature and pH fluctuations) typical of on-skin applications. Therefore, it is highly desirable to develop a robust cost-effective and highly sensitive cortisol sensor.

Molecularly imprinted polymers (MIPs) aim at utilizing a biomimetic approach to generate three-dimensional cavities within a polymer network. MIPs are sterically and chemically complementary to a target analyte, thereby conferring a molecular recognition capability analogous to that of biological antibodies. In contrast to conventional biological recognition elements, such as antibodies, MIPs feature superior tailorability, cost-effectiveness, and high stability under extreme environmental conditions. When integrated into electrochemical sensing platforms, MIPs enable precise detection through high-affinity binding with the target analyte [18,19,20,21].

The development of cortisol-specific nucleic acid aptamers was first reported by Martin J.A. et al. in 2014 [22], followed by the first aptamer-based sensor for cortisol detection by Sanghavi B.J. et al. in 2016 [23]. Concurrently, Bhansali et al. invented the first reusable electrochemical MIP sensor for cortisol detection [24]. Parlak et al. developed a wearable sensing patch for real-time cortisol assessment in sweat during physical activity. This device integrates an organic electrochemical transistor (OECT) with a flexible microfluidic system on a flexible and stretchable substrate [25]. The microfluidic system collects and transports sweat to the sensing interface. The device quantifies cortisol by measuring current changes upon selective binding, with a detection range of 0.01–10 mM, a response time under 1 min, and a limit of detection (LOD) of 1 pg/mL. More recently, Madhu et al. fabricated a non-adhesive, superhydrophilic zinc oxide nanorod electrochemical immunosensor on a flexible carbon fiber substrate for sweat cortisol analysis [26]. This textile-based design remarkably enhances user comfort and wearability. However, a majority of previously reported devices are hampered by restrictions such as complex fabrication protocols, high analyte concentration requirements (typically in the mM range), substantial costs, and susceptibility to auto-oxidation. These limitations dramatically worsen the electrochemical performance and stability of the sensors. Therefore, it is highly desired to develop a highly sensitive, low-cost and simple electrochemical cortisol sensor.

Despite the high selectivity and good stability, the sensing performance of MIP-based sensors is significantly constrained by unmodified electrodes due to low conductivity, sparse surface area, and inferior mass transfer kinetics. To overcome these challenges, the integration of nanomaterials plays a pivotal role in improving sensing behaviors. Benefitting from high surface-to-volume ratios, excellent electrical conductivity and biocompatibility, Gold nanoparticles (AuNPs) are considered ideal candidates. The incorporation of AuNPs significantly improves charge transfer efficiency and amplifies the electrochemical signal, thereby accelerating reaction kinetics and reducing response time [27,28,29,30].

In this study, a high-performance portable electrochemical cortisol sensor (PECS) was developed on the basis of a composite of Prussian blue and a polypyrrole-based molecularly imprinted polymer for the real-time and non-invasive detection of cortisol. Gold nanofilms were fabricated on screen-printed carbon electrodes via electrodeposition. Material characterization was conducted using scanning electron microscopy (SEM) and energy-dispersive X-ray spectroscopy (EDS). Furthermore, the dependence of sensing performance on polymerization conditions and gold film thickness were systematically investigated. The as-prepared sensor demonstrates a wide and linear detection range from 1 fM to 1 µM, an ultra-low detection limit of 0.4112 fM, and a high sensitivity of 15.518 nA∙lg(nM^−1^)∙cm^−2^. The PECS also exhibits exemplary selectivity, reproducibility, and long-term stability for cortisol detection. This work provides a new possibility for developing soft bioelectronics for non-invasive, real-time mental health monitoring.

## 2. Experimental

### 2.1. Materials

The experimental materials were sourced from various suppliers. Ultrapure water (≥18.2 MΩ·cm) came from Sichuan Youpu Ultrapure Technology Co., Ltd. (Chengdu, China). Anhydrous ethanol (AR ≥ 99.7%), hydrochloric acid (AR ≥ 99.7%), potassium chloride (AR, 99.5%), potassium ferricyanide (AR), potassium ferrocyanide (AR), anhydrous glucose (AR), and ascorbic acid (AR, 99.7%) were obtained from Chengdu Cologne Chemicals Co., Ltd. (Chengdu, China). Gold chloride was provided by Lvyuan Company (Nantong, China), while phosphate-buffered saline (PBS, dry powder, pH 7.2–7.4) was supplied by Biosharp (Nantong, China). Cortisol, glycineI (≥98.5%), dopamine (98%), progesterone (98%), pyrrole (99%), and Prussian blue (BS) were purchased from Shanghai Macklin Biochemical Co., Ltd. (Shanghai, China).

### 2.2. Electrodeposited Gold

First, a 0.0033 M chloroauric acid stock solution was prepared by dissolving 1 g of HAuCl_4_ in deionized water and diluting to 100 mL. Screen-Printed Carbon Electrode (SPCE) was then subjected to chronoamperometry (+1.2 V, 180 s) in saturated Na_2_CO_3_ under magnetic stirring (300 rpm). The electrode was then dried in the dark for 12 h. Gold was subsequently electrodeposited from a 0.5 mM HAuCl_4_ solution using cyclic voltammetry (10 cycles, +0.2 to −1.0 V, 50 mV/s). Rinsing with ultrapure water 3 times, produced the final Au/SPCE.

### 2.3. Prepare the Pre-Polymerization Solution

The pre-polymerization solution for molecularly imprinted polymer (MIP) fabrication was formulated to contain a pyrrole (Py) monomer concentration of 20 mM. The template molecule, cortisol, was then introduced at various molar ratios relative to the Py monomer (i.e., 10:5, 10:4, 10:3, 10:2, and 10:1). This was performed in a phosphate-buffered saline (PBS) solution containing 5 mM Prussian blue (PB), 10 mM FeCl_3_, 10 mM K_3_[Fe(CN)_6_], 1 mM HCl, and 1 mM KCl. The resulting mixture was subjected to ultrasonication for 30 min, followed by stirring for 1 h at ambient temperature, to ensure complete homogenization and yield the final polymerization precursor solution.

### 2.4. Electropolymerization and Elution

A three-electrode system was used, consisting of an SPCE, a graphite rod (counter), and Ag/AgCl (reference). A molecularly imprinted polymer (MIP) was created on the working electrode through electropolymerization using cyclic voltammetry (CV), scanning between −0.2 and +0.9 V for 5–30 cycles at 50 mV/s. This process formed a polypyrrole network that entrapped Prussian blue nanocubes and cortisol molecules.

After polymerization, the electrode was rinsed. The cortisol template was then removed by electrochemical elution, which involved running CV for 20 cycles between −0.2 V and 0.8 V in a PBS solution.

A non-imprinted polymer (NIP) electrode was created using the exact same procedure, but without adding cortisol during the polymerization step.

### 2.5. Characterization

The microstructural surface morphology of the prepared samples was characterized using field emission scanning electron microscopy (FESEM, GeminiSEM 300, Zeiss, Jena, Germany). The types and contents of the components of the material microregion were analyzed by EDS spectrometer(EDS, Octane Elect Super C5, AMETEK, Shanghai, China).

### 2.6. Measurement Conditions

In this work, unless otherwise specified, the Differential Pulse Voltammetry (DPV) parameters were set with an initial potential of 0 V, a final potential of 0.3 V, an amplitude of 0.05 V, and a sampling interval of 0.01 s. The basic parameters for preparing the sensor are: 20 mM pyrrole, 8 mM cortisol, 5 mM Prussian blue, 15 electropolymerization cycles for sensor preparation, 20 elution cycles, a 0.5 mM chloroauric acid solution, and 5 cycles for gold deposition.

## 3. Results and Discussion

**Figure 2:**
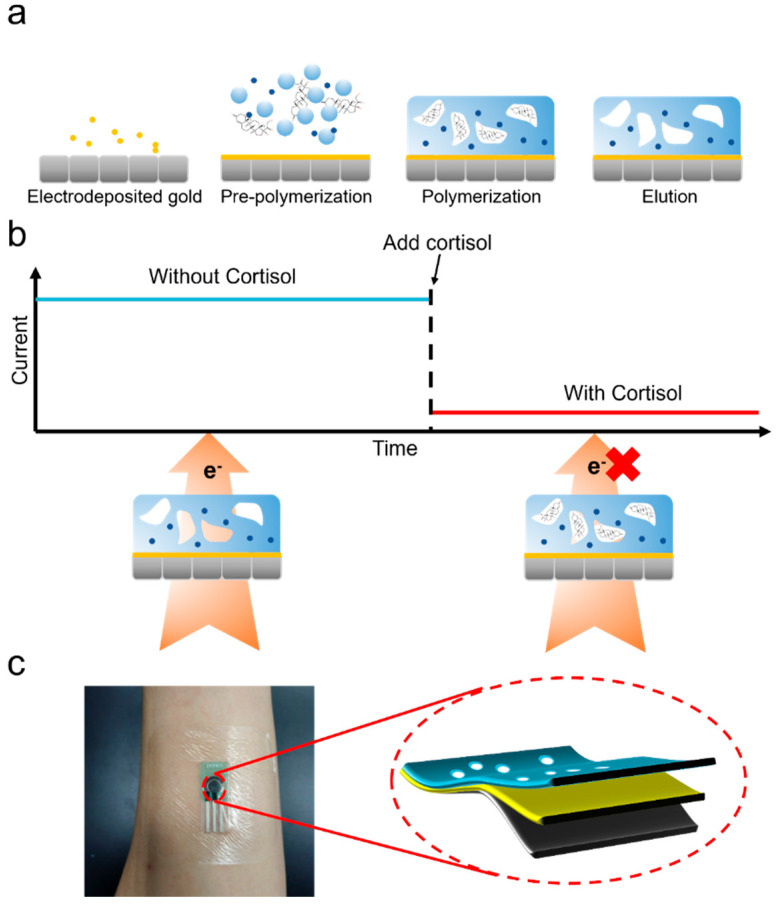
Sensor preparation process. (**a**) The process of gold plating MIP sensors, polymerization, and elution of cortisol; (**b**) Changes in sensor current after an increase in cortisol concentration; (**c**) Thel image of the prepared sensor.

Figure 2a illustrates the fabrication process of the Molecularly Imprinted Polymer (MIP), which comprises four primary steps: gold electrodeposition, pre-polymerization solution preparation, electropolymerization, and template elution. The detection mechanism is depicted in Figure 2b. In the absence of cortisol, the Prussian blue redox probe generates a high current signal. The specific binding between cortisol molecules and the imprinted cavities hindered the electron transfer and output current. This inverse relationship between cortisol concentration and current intensity enables specific quantification. The portable electrochemical cortisol sensor (PECS) is designed for direct application to human skin by adopting a commercial screen-printed carbon electrode (SPCE) (Figure 2c).

**Figure 3:**
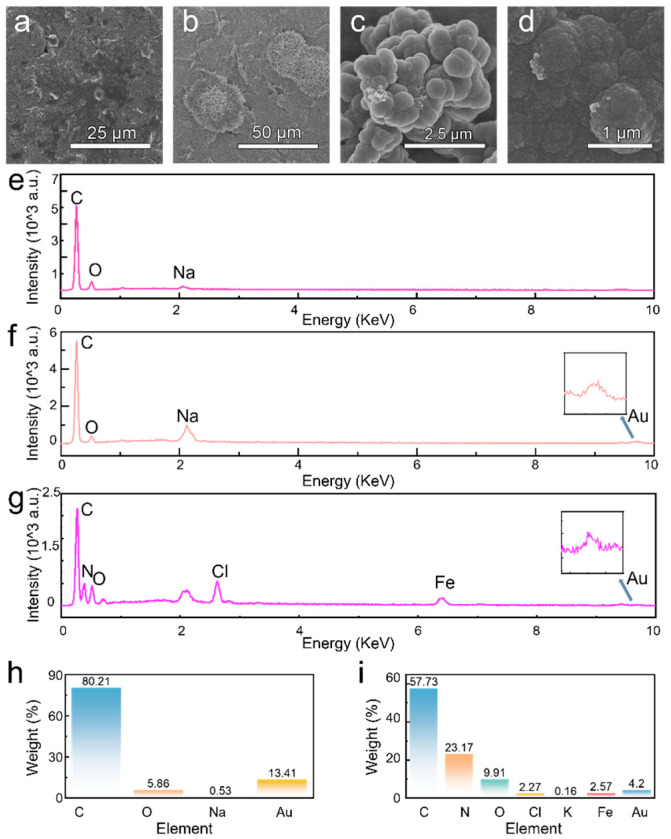
SEM and EDS of MIP sensor. (**a**) Surface of the cleaned bare electrode; (**b**) Surface after electroplating with gold; (**c**) Surface after electro-polymerization; (**d**) Surface after elution; (**e**) EDS of the cleaned bare electrode; (**f**) EDS of the electrode after electroplating with gold; (**g**) EDS of the electrode after elution; (**h**) Mass percentage of elements on the electrode surface after electro-deposition of gold; (**i**) Mass percentage of elements on the electrode surface after elution.

Scanning electron microscopy (SEM) was utilized to characterize the morphologies of the prepared electrode surface at various fabrication stage (Figure 3a–d). Clearly, the electrochemical deposition forms a dense gold film with some aggregated and block-like structures (Figure 3b), confirming the successful gold modification on bare SPCE. Then, the polymerization of polypyrrole and subsequent elution of the cortisol template give rise to a granular surface of polypyrrole film (Figure 3c,d). It is worth noting that the overoxidation process renders rough and porous microstructure with three-dimensional molecularly imprinted cavities.

The EDS analysis confirms the step-by-step modification of the electrode surface. Initially, the bare electrode (Figure 3e) encompasses carbon and oxygen and trace-level sodium residues from the pretreatment process, confirming a clean and uncontaminated carbon substrate. After gold plating (Figure 3f,h), both the strong gold peak and corresponding high mass percentage were observed, validating the successful deposition of gold particles. Finally, after the synthesis of the molecularly imprinted polymer (Figure 3g,i), a distinct nitrogen peak—a key component of polypyrrole—provides direct evidence that the polymer was successfully formed on the electrode surface.

**Figure 4:**
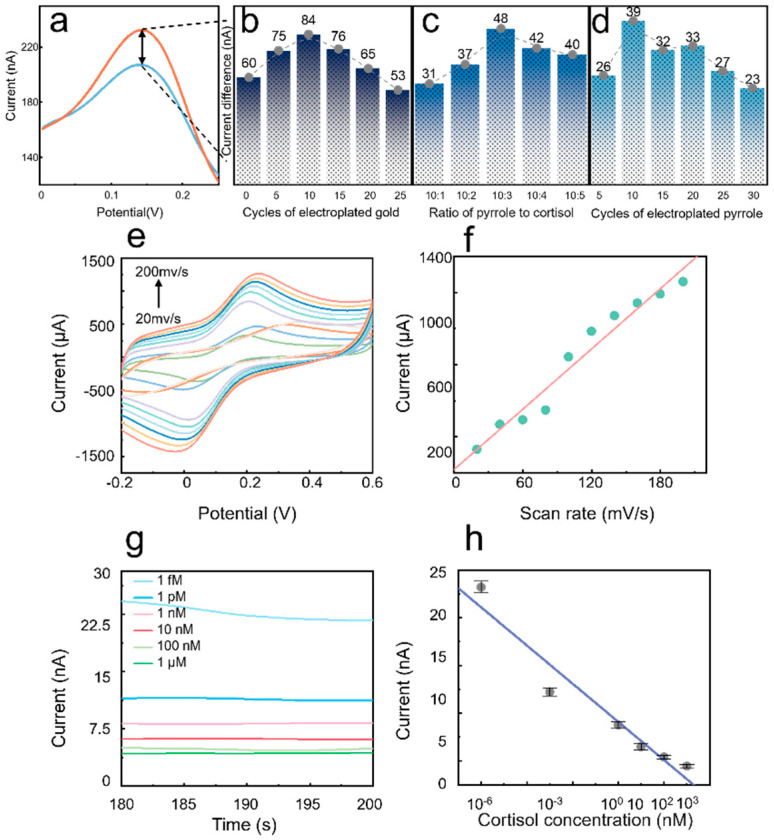
Comprehensive Analysis of MIP sensor. (**a**) The DPV response of the sensor in 1 μM cortisol solution and pure PBS solution; (**b**) The difference in DPV peak current of the sensors in 1 μM cortisol and pure PBS solution after different cycles of electroplated gold; (**c**) The difference in DPV peak current of the sensor after polymerization at different concentrations of pyrrole and cortisol in 1μM cortisol and pure PBS solution. (**d**) The difference in DPV peak current of the sensor in 1μM cortisol and pure PBS solution after different cycles of electropolymerization; (**e**) Cyclic voltammograms of MIP sensors in the electrolyte (scan rate from 20 mV/s to 200 mV/s); (**f**) The oxidation current peaks and their fitting curves corresponding to different scanning speeds; (**g**) The constant potential-timed current curve of MIPs sensors in electrolytic solution (cortisol concentration from 1 μM to 1 fM); (**h**) Fitting curve of the sensor stable current.

To achieve the optimal performance of the sensor, modulation of several key parameters in the fabrication process of PECS was conducted. As shown in Figure 4a, sensors prepared with different parameters were tested using differential pulse voltammetry (DPV) in both a 1 μM cortisol PBS solution and a pure PBS solution. A larger current difference hints a better cortisol detection performance for the sensor. Figure 4b shows the influence of cycle number during gold plating performance on the current difference. Notably, the sensing performance undulates with cycle number and maximizes at 10 cycles. This is because that insufficient gold nanoparticles limited the signal transduction, while excessive gold quantity achieves a high current baseline and thus diminishes current variation rate upon cortisol adsorption.

Figure 4c shows the dependence of MIP sensor performance on the molar ratios of pyrrole. Apparently, a ratio of 10:3 yielded the optimal sensing performance. Figure 4d displays the sensing performance of PECS fabricated with various number of polypyrrole electropolymerization cycles. The optimal performance was found at 10 cycles. This is because with too few polymerization cycles, there are insufficient attachment sites for the template molecules (cortisol), leading to unsuccessful imprinting and fewer cavities. This results in an insufficient barrier to electron transfer upon cortisol binding, causing a small change in potential. Conversely, when the number of polymerization cycles is too high, the polypyrrole film becomes too thick. This blocks most of the cavities and makes it difficult for cortisol molecules to be eluted from or bind with them, which also leads to a small change in potential.

To elucidate the electrochemical kinetics at the modified electrode interfaces, the influence of varying scan rates (*ν*) on the redox behavior of a standard probe was investigated using cyclic voltammetry (CV). As depicted in Figure 4e, both the anodic and cathodic peak currents are proportional to the scan rate. Concurrently, the anodic peak potential shifted positively, while the cathodic peak potential shifted negatively. This peak-to-peak separation upon increasing scan rate originates from a quasi-reversible electron transfer process at the electrode surface. As plotted in Figure 4f, the anodic peak current exhibited a strong linear relationship with the scan rate (*ν*) with a high correlation coefficient (R^2^ = 0.9537). This linear dependence of peak current on *ν* (rather than on the square root of the scan rate, *ν*^1/2^) confirms that the surface-controlled (adsorption) mechanism rather than diffusion dominates the redox process of the probe at the MIP interface [31], the heterogeneous electron transfer rate constant (k0) based on the Laviron equation is 2.2 cm·s^−1^, which confirms the quasi-reversible nature of the electrochemical process [32,33].

A sensor with optimal performance was fabricated using the previously determined parameters, and chronoamperometry was used to test the steady-state current at different concentrations of cortisol (Figure 4g). Each MIP-modified electrode was incubated for 10 min in PBS solutions containing various cortisol concentrations to ensure sufficient time for the analyte to bind with the imprinted cavities. After incubation, the chronoamperometric response was recorded for 200 s at an applied potential of +0.1 V. The relationship between the steady-state current and cortisol concentration was established by plotting the steady-state current against the logarithm of the concentration (log *C*), as shown in Figure 4h. The data exhibited a strong linear correlation across a wide dynamic range with a high correlation coefficient of R^2^ = 0.93854 and a sensitivity of 15.158 nA∙(log nM)^−1^∙cm^−2^.

Furthermore, the limit of detection (LOD) was calculated to be 0.4112 pM based on the IUPAC criterion (LOD = 3σ/S) Importantly, the sensor’s operational linear range (1 fM–1000 nM) effectively covers the physiological concentration of cortisol found in human sweat under resting conditions (approx. 22.07–389.11 nM [34]). This ultralow detection limit, combined with the sensor’s advantages of miniaturization and low power consumption (<0.5 V) demonstrate significant potential for real-time wearable sweat cortisol monitoring [35,36].

Note that this operational linear range (equivalent to 1 fM–1000 nM) effectively covers the physiological concentration of cortisol reported in human sweat under resting conditions. Notably, this ultralow detection limit (0.4112 pM) is approximately five orders of magnitude lower than the basal physiological concentration (~23.7 nM), providing an ample signal-to-noise margin for trace-level detection. This outstanding analytical performance, combined with the sensor’s inherent advantages of miniaturization and low power consumption (operating voltage < 0.5 V), underscores its significant potential for integration into wearable, real-time sweat cortisol monitoring systems.

**Figure 5:**
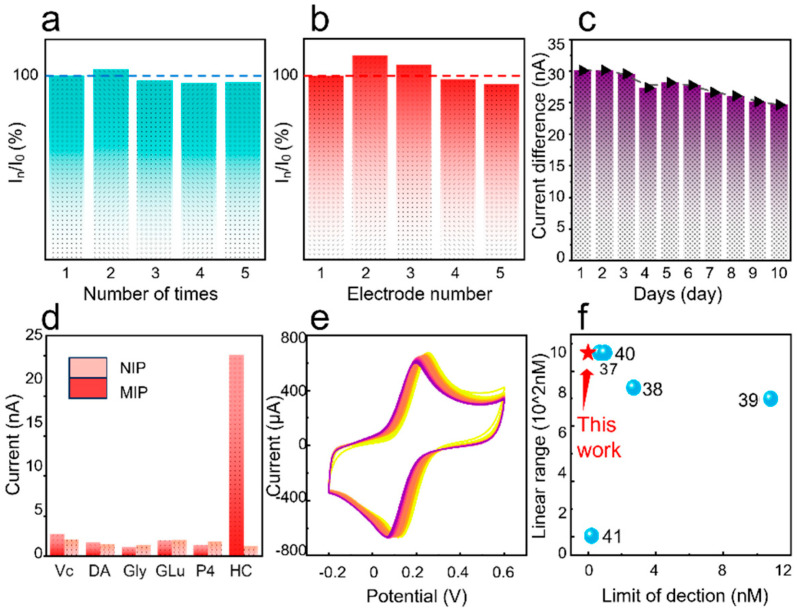
Comprehensive Analysis of MIP cortisol sensor. (**a**) Bar chart of the ratio of I-t current magnitudes after testing the same device five times for stability; (**b**) Bar chart of the ratio of I-t current magnitude after different devices stabilize; (**c**) Ten-day long-term stability test of the sensor; (**d**) Specificity tests of MIP sensors and NIP sensors for different substances; (**e**) Sensor 50 times CV cycle test curve; (**f**) Comparison of performance with other cortisol sensors [37,38,39,40,41].

Figure 5a presents the reproducibility of the PECS under 1 µM cortisol in five successive trials. These measurements yielded a low relative standard deviation (RSD) of 2.85%, validating the high repeatability of the as-prepared sensor. In Figure 5a, $I_{0}$ represents the steady-state current value obtained after the first test, which is set as the reference. $I_{n}$ denotes the steady-state current value obtained after different tests of the same electrode. To evaluate the batch-to-batch consistency of the fabrication protocol, five independently fabricated MIP sensors were tested under identical chronoamperometric conditions by measuring its response to a 1 µM cortisol solution at an applied potential of +0.1 V. In Figure 5b, $I_{0}$ represents the steady-state current value obtained from the first electrode after the test, which is set as the reference. $I_{n}$ denotes the steady-state current value obtained from different electrodes. A statistical analysis of their respective steady-state currents (Figure 5b) yielded a relative standard deviation (RSD) less than 6.2%. This low RSD value signifies excellent inter-batch reproducibility, indicative of robustness and reliability for scalable production.

To evaluate the long-term stability of the sensor, a 10-day continuous monitoring experiment was conducted. The protocol involved daily measurements with the same molecularly imprinted sensor, recording the difference in steady-state current between a 1 μM cortisol solution and a blank PBS solution under standard testing conditions (10 mM PBS, pH 7.4, 25 °C), the sensor was tested once each day during the same time period. As presented in Figure 5c, the sensor retained 85% of its initial current response after 10 days of usage, with an average daily decay of only 1.5%. This good stability further validates the robust, cross-linked structure of the molecularly imprinted polymer. The observed minor signal degradation (15%) comes from the slow oxidation of the polypyrrole film and the gradual aging of the screen-printed electrode upon prolonged exposure to air. This finding reveals the long-term stability for continuous monitoring applications in wearable and portable devices.

The chronoamperometric responses of both the MIP and a non-imprinted polymer (NIP) control sensor were recorded at an applied potential of +0.1 V (vs. Ag/AgCl) toward target analyte (cortisol) and other interferent at a constant concentration of 1 μM. The results, presented in Figure 5d, demonstrate that the MIP sensor exhibited a significantly higher current response to cortisol compared to those of other interferents. In contrast, the NIP control sensor—devoid of specific recognition sites—exhibited negligible and non-selective responses toward both cortisol and the interfering species. This excellent selectivity confirms that the imprinted cavities of MIPs are conducive to specific analyte recognition.

Figure 5e shows the cyclic voltammetry curves of the prepared MIP sensor after 50 cycles of testing in a deionized water solution containing 5.0 mM K_3_[Fe(CN)_6_]/K_4_[Fe(CN)_6_] and 0.1 M KCl. It can be clearly seen that the cyclic voltammetry curves from the 50 measurements overlap well with each other with negligible deviation, indicative of great repeatability. In comparison with other reported works (Figure 5f), the linear detection range and detection limit of as-prepared PECS obviously overwhelm those of other reported sensors [37,38,39,40,41]. This superiority attains the capability of the proposed sensor for trace-level biofluid detection. Table 1 lists more detailed information on various cortisol sensors, demonstrating the excellent performance of the sensor developed in this work. The ultra-low detection limit achieved in this work may result from the synergistic enhancement of multiple effects. Gold nanoparticles significantly improve surface conductivity, while Prussian blue, as an efficient redox mediator, forms rapid electron transfer pathways with the conductive polypyrrole network. Meanwhile, by optimizing the thickness of the MIP film and the ratio of monomer to template, highly accessible recognition sites are ensured, and the signal response is maximized when the target binds. Combined with low-noise constant current detection conditions, these synergistic effects significantly enhance the signal-to-noise ratio, boosting the detection sensitivity by three orders of magnitude compared to previous MIP-based sensors.

## 4. Conclusions

In conclusion, a high-performance portable electrochemical cortisol sensor (PECS) was successfully developed based on molecularly imprinted polymers (MIPs) utilizing a gold-nanoparticle-modified screen-printed carbon electrode. The fabricated sensor exhibited excellent sensitivity, high selectivity, good reproducibility, and remarkable long-term stability. The fabricated PECS features a wide linear detection range from 1 fM to 1 μM and an ultra-low detection limit of 0.4112 fM, enabling the accurate quantification of cortisol in sweat. The high specificity of the MIPs recognition sites together with the low cost and straightforward fabrication process pave the way for the real-time and non-invasive monitoring of psychological stress. This work provides a new possibility for the development of advanced wearable and portable devices for mental health monitoring.

## Figures and Tables

**Table 1:** The comparison of sensing performance.

Sensing Platform	Sensing Molecule	Linear Range	LOD	References
Ag@AgO/PANI	Anti-C_mab_	1 pM–1 μM	0.64 pM	[42]
MIP-PPy-SPCE	MIP	1 pM–10 μM	1 pM	[25]
MIP-PPy-SPCE	MIP	1 nM–1 μM	0.9 nM	[29]
Au@PDMS	Anti-C_mab_	1 pM–1 μM	0.2 pM	[11]
GO-SiC	Anti-C_mab_	100 fg/mL–1 μg/mL	90 fg/mL	[43]
Ti-Cu BMOFs/MIPs	MIP	0.05 nM–1 μM	37 pM	[44]
MIP-PPy-Au@SPCE	MIP	1 fM–1 μM	0.4112 fM	This work

## Data Availability

The data are available from the corresponding author on reasonable request.
